# Choroidal and retinal vascular changes in adults with Down syndrome: Insights into the Alzheimer's disease continuum

**DOI:** 10.1002/alz.70228

**Published:** 2025-05-12

**Authors:** Jamie Mitchell, Adam Threlfall, Kenneth Sloan, Luke Smyth, Jessica Beresford‐Webb, Madeleine J. Walpert, Tunde Peto, Tom MacGillivray, Antony Holland, Imre Lengyel, Lajos Csincsik

**Affiliations:** ^1^ Wellcome‐Wolfson Institute for Experimental Medicine School of Medicine Dentistry and Biomedical Sciences Queen's University Belfast Belfast UK; ^2^ Centre for Clinical Brain Sciences University of Edinburgh Edinburgh UK; ^3^ Department of Computer Science University of Alabama at Birmingham Birmingham Alabama USA; ^4^ University of Cambridge, The Old Schools Cambridge UK; ^5^ Centre for Public Health, School of Medicine Dentistry and Biomedical Sciences Queen's University Belfast, Institute of Clinical Science Block A, Royal Victoria Hospital Belfast UK

**Keywords:** choroidal vascularity index, choroidal vasculature, CVI, Down syndrome, fractal dimension, multimodal retinal imaging, OCT, optical coherence tomography, retinal microvasculature, retinal vasculature, tortuosity, ultra‐widefield imaging, width gradient

## Abstract

**INTRODUCTION:**

Retinal and choroidal vascular changes have been proposed as a non‐invasive central nervous system (CNS) proxy for clinical trials in Alzheimer's disease (AD). However, their role in Down syndrome (DS), the largest genetically predisposed group for AD, remains unclear.

**METHODS:**

We conducted ultra‐widefield and optical coherence tomography imaging on 24 individuals with DS and 17 controls and extracted various vascular parameters. Data were analyzed using logistic and linear regression models.

**RESULTS:**

The DS retinae exhibited supernumerary vessels that were wider and thinned more rapidly along their paths (*p* = 0.01). There was a more complex central retinal (*p* = 0.047) and a less complex peripheral retinal vascular tree (*p* = 0.001), with increased numbers of peripheral microvascular abnormalities (*p* = 0.038) and reduced choroid vascularity index in DS (*p* < 0.001).

**DISCUSSION:**

We found that retinal and choroidal vascular changes are present in DS adults before the clinical onset of AD and might become early surrogates for cerebral vascular abnormalities.

**Highlights:**

- Eye imaging in DS showed retinal and choroidal changes seen in AD.
- Far peripheral retinal microaneurysms and hemorrhages associated to DS.
- Wider and faster thinning vessels associated to DS.
- Reduced vascular tree complexity in the peripheral retina linked to DS.
- Lower choroidal vascularity associated with DS.

## 1 BACKGROUND

Alterations in the retinal vasculature, characterized by changes in vessel caliber, the complexity of the retinal vascular tree (fractal dimension [FD]), and the degree of vessel thinning along the vessel path (width gradient [WG]), have been associated with mild cognitive impairment (MCI) due to Alzheimer's disease (AD) and sporadic AD (sAD).^1^, ^2^, ^3^, ^4^, ^5^ In addition, a diminished choroidal thickness and vascularity (represented as choroidal vascularity index [CVI]) has been noted in cases of MCI due to AD and sAD.^6^, ^7^ These vascular changes may serve as a noninvasive, cost‐effective proxy for the central nervous system (CNS) that could be employed in early clinical trials to monitor disease progression and/or treatment efficacy.

Down syndrome (DS) is the largest genetically predisposed population to develop AD,^8^ but our understanding of retinal and choroidal vasculature in DS and the changes associated with the sAD continuum (MCI and sAD) remains limited. The only published literature focuses exclusively on the presence of supernumerary vessels in DS at the optic disk, resembling the spokes of a wheel (spoke‐like pattern) compared to adults without DS controls, and this has been attributed to altered retinal development during embryogenesis.^9^, ^10^, ^11^ However, our group has reported thickening of the retina and choroid in adults with DS before the onset of clinical dementia^12^, ^13^ that was not observed in children and younger adults with DS.^14^, ^15^ This temporality of changes might suggest that it may be an early sign of an inflammatory event,^12^ potentially secondary to amyloid deposition in the retina and choroid that has been shown in human *post mortem* tissue of AD donors^16^ or in mouse and rat models of AD.^17^, ^18^

We focused on adults with DS with no clinical signs of dementia but who were within the age range where cerebral AD pathology begins developing or has developed.^19^

Using quick, noninvasive, inexpensive, and well‐tolerated multimodal eye imaging, we aimed to explore the potential of retinal and choroidal vasculature as a preclinical proxy for cerebral AD pathology in this patient population.

## 2 METHODS

### 2.1 Study design and recruitment

Participants with DS (27–53 years of age) were recruited from an established DS cohort in Cambridge, UK, while adults without DS (control) (22–51 years of age) were locally recruited through the University of Cambridge and age‐matched to the DS group.^12^ The study received ethical approval from the East of England Cambridge Central Research Ethics Committee (study ref. 14/EE/1118) and was conducted in accordance with the World Medical Association Declaration of Helsinki. Written informed consent was obtained from all participants. For individuals with DS who lacked the capacity to consent, advice was sought from an identified consultee in accordance with the Mental Capacity Act 2005.

**RESEARCH IN CONTEXT**

1. **Systematic review**: According to our literature review using PubMed, supernumerary retinal vessels with spoke‐like pattern has already been described in people with Down syndrome (DS). We could not identify studies exploring vascular changes in the eye in relation to dementia in this patient population.
2. **Interpretation**: Using rapid, non‐invasive multimodal eye imaging, including ultra‐widefield imaging, we identified retinal and choroidal vascular changes in individuals with DS before clinical dementia onset, similar to sporadic Alzheimer's disease (sAD). We also observed peripheral retinal microaneurysms and hemorrhages, previously unreported in this population. These unique retinal changes highlight the potential of accessible ocular biomarkers for monitoring disease progression and stratifying patients for treatment trials.
3. **Future directions**: These retinal and choroidal vascular changes in DS should be correlated with brain imaging, fluid biomarkers, and clinical measures over time.

After pharmacological pupil dilation, both eyes of a participant were imaged using the ultra widefield (UWF) scanning laser ophthalmoscope (SLO) (Dunfermline, Optos plc, UK) and optical coherence tomography (OCT; Heidelberg Engineering GmbH, Heidelberg, Germany, Camera Model S3610). The Cambridge Cognition Examination DS (CAMCOG‐DS) was used to assess areas of cognition known to decline with the onset of dementia.^20^ The level of intellectual disability was stratified according to the Diagnostic and Statistical Manual of Mental Disorders (fifth edition) as mild and moderate.^21^ The exclusion criteria were a history of eye surgery within 3 months of retinal imaging, intravitreal injection, severe cataract, glaucoma, age‐related macular degeneration, and diabetes mellitus. A further exclusion criterion was the presence of a neuropsychiatric illness other than AD. While control participants did not undergo the CAMCOG‐DS evaluation, dementia diagnosis was an exclusion criterion in this group.

### 2.2 Analysis of retinal vasculature

#### 2.2.1 Quantitative retinal vascular parameters–OCT

##### Peripapillary vessel number

**FIGURE 1:**
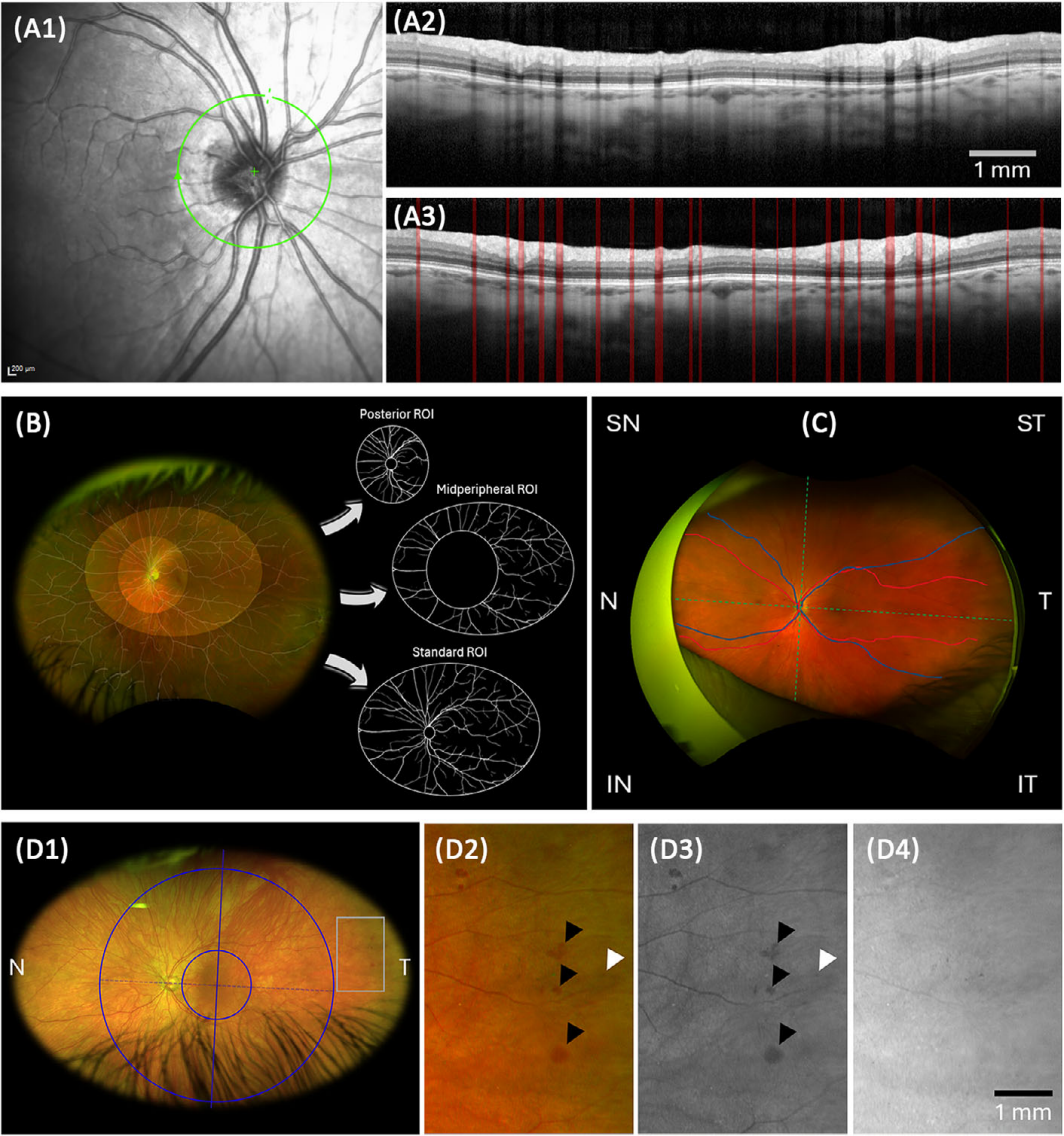
Retinal vascular parameters. An infrared fundus image (A1) displays a green ring with a 3.4 mm diameter, marking the scanning area centered on the optic disk. The corresponding OCT B‐scan (A2) shows horizontal shadows created by blood vessels in the inner retinal layers. Each of these vessel‐related shadows is segmented in red (A3) using the OCTseg software. FD was extracted using standardized ROI on an ultra‐widefield false color image, further divided into posterior and midperipheral ROIs (B). Vessel WG, WI, and tortuosity was measured for selected major arterioles (red) and venules (blue) in each quadrant (divided by the horizontal and vertical dashed lines centered on the fovea and aligned to the foveal‐optic disk axis), and the values were averaged to create temporal, nasal (divided by the vertical dashed green line), and global values (incorporating the entire image) (C). Qualitative retinal vascular parameters were extracted using the Moorfields grading grid (blue lines and circles) from false‐color composite ultra‐widefield images (D1,2), which consist of green (D3) and red channels (D4). The area within the grey rectangle, showing microaneurysm (white arrowhead) and hemorrhage (black arrowhead), is magnified in panels D2–4. These microvascular abnormalities are visible only in the composite (D2) and green channel (D3) views, indicating that the pathology affects the retinal blood vessels and not deeper layers of the eye, such as the choroidal vessels. FD, fractal dimension; IN, inferonasal; IT, inferotemporal; N, nasal; OCT, optical coherence tomography; ROI, region of interest; SN; superonasal; ST, superotemporal; T, temporal; WG, width gradient; WI, width intercept

Optic nerve head (ONH) OCT scans from 38 eyes of 21 individuals with DS, and 33 eyes of 17 control participants were analyzed after an initial quality control process (Figure S1B). This process excluded off‐centered scans and those with poor signal quality. The scans were then segmented to determine the number of vessels. The OCT scans were ring scans with a 3.4 mm diameter, centered on the optic disc, and performed using automated real‐time (ART) averaging 100 frames per scan (Figure 1A). We employed OCTseg software to automatically segment retinal blood vessels based on the shadows they cast on the scan^22^ (Figure 1A). Manual corrections to the segmentation were performed by a masked operator (J.M.) to address errors, such as unsegmented vessels, misclassified shadows, and merged vessels. Very thin ciliary vessels, measuring 1 to 2 pixels in width (approximately 6.8–13.6 µm), were excluded from analysis due to frequent omission by the algorithm. Eyes were categorized into two groups based on the number of vessels detected on the B‐scan: low (< 18 vessels) and high (≥18 vessels). The threshold of 18 vessels was established according to previous research by Jaeger.^23^

#### 2.2.2 Quantitative retinal vascular parameters–UWF

After quality control (Figure S1A), UWF false‐color images of 33 eyes of 18 DS and 34 eyes of 17 control participants were included in the retinal vascular analysis (Figure 1B,C). Off‐centered images and images with artifacts, such as eyelashes or eyelids, as well as obscured or blurred vessels due to media opacities, were excluded from the study.

The retinal vasculature was automatically segmented and classified into arterioles and venules using the VAMPIRE‐UWF (Vasculature Assessment Platform for Images of the Retina; Universities of Edinburgh and Dundee, UK) software and segmentation and/or classification error manually corrected if needed by a trained operator (J.M.). Measures such as FD, representing the branching complexity of the retinal vascular tree; WG, indicating how the vessel narrows along its path; width intercept (WI), estimating vessel caliber at the optic disc edge (a proxy for central retinal artery and vein equivalent [CRAE and CRVE]); and tortuosity (TORT), quantifying how much a vessel twists and turns, were calculated.

##### FD

A standard region of interest (ROI) was used for fractal extraction, excluding the area encompassing the optic disc and aligned based on the optic disc‐fovea axis (Figure 1B). This standard ROI, measuring 319 mm^2^, was previously developed by Pead et al. to cover the maximum average retinal area free from artifacts or obstructions, such as eyelashes or eyelids, which can obscure the peripheral retina.^2^ The standard ROI was then divided into two sub‐regions: a posterior ROI centered on the disc and a mid‐periphery ROI, which includes the remaining area beyond the posterior ROI and centered on the fovea (Figure 1B).^2^ The posterior ROI was defined as a ring extending three optic disc diameters from the edge of the optic disc, with a radius of 5.2 mm and a field of view of approximately 50°. The mid‐peripheral ROI has a vertical field of view of approximately 90° and a minor radius of 8.5 mm. The FD of the retinal vasculature was calculated using the sandbox count method for each ROI listed above^24^ (Figure 1B). Total vascular FD, encompassing both arterioles and venules, was calculated, along with separate FDs for arterioles (FDa) and venules (FDv).

##### WG, WI, and TORT

WG was calculated for the main vessel paths in each quadrant by recoding their caliber at regular intervals along the vessel path considering the entire image (Figure 1C) and computing the gradient of thinning using a fitted robust regression line.^3^, ^25^ The WI was derived using the Y‐intercept of the same regression line, which predicts the vessel caliber at the optic disk edge and has previously been described.^3^ The tortuosity density (TORT) was calculated using the same vessel paths employed for the WG calculation considering the entire image (Figure 1C). This measure was determined by evaluating the total number of curves (separated by inflection points) within a smoothed vessel path and the total deviation of each curve from a straight path.^26^, ^27^

The selected vessel paths for WG and TORT calculations were the largest and longest vessels in each quadrant that were not obscured or fragmented by poor image quality (Figure 1C).

#### 2.2.3 Qualitative retinal vascular parameters–UWF

##### Retinal microvascular abnormalities

Following initial quality control (Figure S1A), UWF false‐color images of 33 eyes from 18 individuals with DS and 34 eyes from 17 control participants were manually assessed for retinal microvascular abnormalities, including microaneurysms and hemorrhages, by a masked, certified grader (L.C.) (Figure 1D1–4). Microaneurysms were identified as small, well‐rounded red dots, while hemorrhages appeared as red patches, which varied in shape and size and were located near retinal vessels. The absence of these features on the red channel of the image (Figure 1D4) indicated that the pathology was confined to the retina and not present in deeper layers, such as the choroid. Ambiguous cases were reviewed with the assistance of a senior ophthalmologist (T.P.), who was masked to participant status, to reach a final decision.

The presence or absence of the described retinal microvascular changes were recorded during the grading process, including their locations across the entire visible retinal surface. To delineate the location of the pathology, the foveal‐centered Moorfields grid–described elsewhere^28^‐was employed, categorizing lesions into central (posterior pole), mid‐peripheral, and far‐peripheral zones, further divided into temporal and nasal hemispheres (Figure 1D1).

An eye was graded positive for microvascular abnormality if it exhibited either microaneurysm or hemorrhage, or both. Subsequently, a hierarchical phenotyping approach was employed, categorizing eyes into two groups based on the type of microvascular abnormality. Eyes exhibiting only microaneurysms were assigned to the microaneurysm group, while eyes with both microaneurysms and hemorrhages, or only hemorrhages, were placed in the hemorrhage group.

### 2.3 Analysis of choroidal vasculature

#### 2.3.1 Quantitative choroidal vascular parameters–OCT

##### CVI

OCT scans of 30 eyes of 18 DS and 34 eyes of 17 control participants were included in the CVI analysis after initial quality control (Figure S1). Eyes with OCT scans with poor signal and/or clipping artifacts were excluded from data extraction and/or analysis.

**FIGURE 2:**
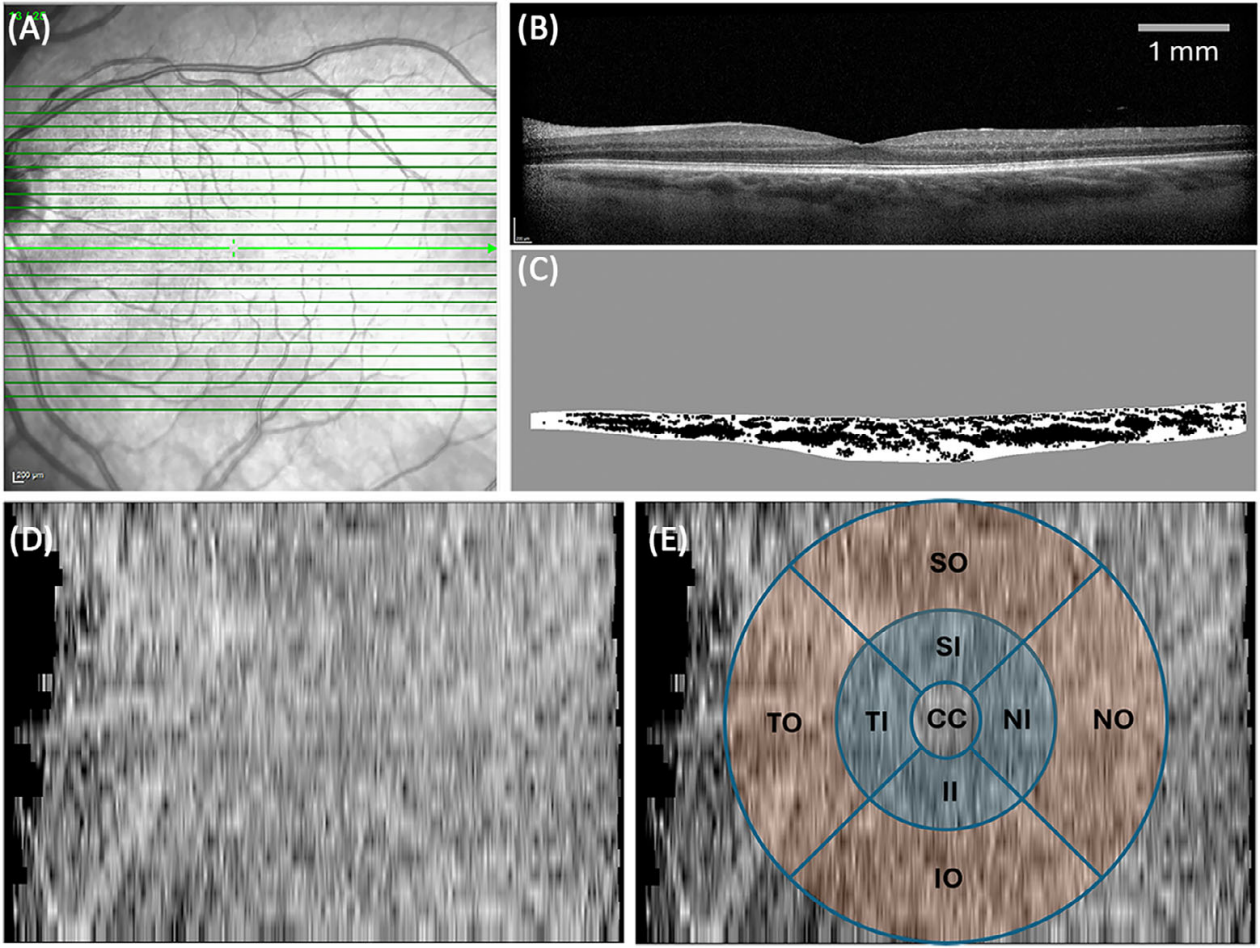
Choroidal vascularity. (A) Infrared fundus image with green lines demarcating the scanning area. (B) One of the 25 corresponding EDI OCT B‐scans (13/25). (C) Cropped and binarized choroid. After all 25 B‐scans underwent cropping and binarization, a choroidal map was created (D), and mean CVI values were extracted for each sector of the ETDRS grid (E). CC, central circle; EDI, enhanced depth imaging; IO, inferior outer; IR, inner ring; NO, nasal outer; OCT, optical coherence tomography; OR, outer ring; TI, temporal inner; SI, superior inner; NI, nasal inner; II, inferior inner; SO, superior outer; TO, temporal outer.

A 30 × 20 OCT volume scan centered on the fovea, acquired in enhanced depth imaging mode (EDI OCT), with 25 B‐scans per volume and 1536 A‐scans (high resolution) per B‐scan, and an ART of 9 was used for CVI extraction (Figure 2A,B). If the EDI OCT scan was unavailable or of insufficient quality, a 30 × 25 OCT volume scan, acquired with EDI mode off, with 61 B‐scans per volume, 768 A‐scans (high speed) per B‐scan, and an ART of 9 was used instead, if available.

The inner boundary of the choroid (Bruch's membrane) was automatically segmented by the Heidelberg Eye Explorer (Heyex) and corrected manually if a segmentation error occurred. Then, scans were exported using Heyex's XML export function in TIFF format accompanied by metadata in XML format containing coordinates of the choroidal inner boundary and information important for volumetric scan re‐construction in ImageJ (National Institutes of Health, Bethesda, Maryland, USA). CVI was extracted, following the manual labeling of choroidal outer boundary by a trained operator (J.M.), automated binarization, and creation of a 3D CVI map image using a series of homebuilt ImageJ plugins (plugins available from author K.R.S. on request). The inner border of the sclera, known as the choroidal scleral interface (CSI), was considered the outer boundary of the choroid. When the suprachoroidal layer (SCL)—comprising the hyperreflective suprachoroidal stroma and the hypo‐reflective suprachoroidal space (SCS)—was present, the inner border of the sclera was located posterior to the SCS. In the absence of the SCL, the inner border of the sclera corresponded to the interface between the hypo‐reflective vasculature and the hyperreflective sclera.^12^

The automated binarization and 3D CVI map (Figure 2C,D) creation steps involved Gaussian smoothing (sigma = 0.5 pixels), conversion to an 8‐bit binary image, Otsu auto local thresholding (at multiple scales), and erosion and dilation to remove too small regions. At each scale, dark pixels further from the edges of the choroid than the Otsu radius were accepted. All accepted dark pixels were accumulated into a single image before erosion and dilation. White pixels were accepted as the stromal area (SA), and the dark pixels were accepted as the luminal area (LA), corresponding to the blood vessels (Figure 2C). The CVI, the proportion of the LA to the total choroidal area, was calculated for each sector of the Early Treatment Diabetic Retinopathy Study (ETDRS) grid, a commonly used grid in OCT imaging (Figure 2E).

### 2.4 Statistical analysis

Demographics and cognitive scores were summarized using descriptive statistics. Categorical variables were reported as absolute frequency and percentage, and continuous variables as mean value with standard deviation (SD) and range (minimum and maximum value). Group differences were assessed using Pearson's chi‐squared test and Fisher's exact test for categorical variables, as well as the Wilcoxon rank sum test for continuous variables.

Both eyes of a participant were included in the analysis if available. All quantitative vascular parameters were initially plotted and presented using box‐and‐whisker plots or density plots (retinal vessel count). Subsequently, we employed generalized estimating equation (GEE) logistic and linear regression models to evaluate the relationship between quantitative and qualitative vascular changes and diagnosis. The participant group was the exposure (with control group as the reference arm), and the vascular parameter was the outcome. This analysis accounted for the correlation between eyes within the same patient using an exchangeable correlation matrix and included adjustments for age and sex. As GEEs are sensitive to outliers, extreme values were imputed to the mean of the group (this adjustment was applied only in the analysis of vascular TORT values for a single participant).^29^

To assess the effect of CAMCOG‐DS on vascular parameters in individuals with DS, GEE logistic and linear regression analyses were performed with CAMCOG‐DS as the exposure and vascular parameters as the outcome, adjusting for age and sex. Marginal R‐squared was calculated for models where a significant relationship was observed between exposure and outcome.

The significance level was evaluated at alpha = 0.05 after adjustment for multiple comparison using false discovery rate correction. RStudio (2024.04.2+764) with R version 4.3.2 (2023‐10‐31; Nickname: Eye Holes) was used for data visualization and statistical analyses.

## 3 RESULTS

We included 24 participants with DS and 17 age and sex‐matched controls in our study (Figure S1). The final number of eyes included in each analysis is shown in the flow chart (Figure S1). The mean age was 39 ± 7 years (range: 27–53) for the DS group and 36 ± 9 years (range: 22–51) for the control group. The mean CAMCOG‐DS score for the DS group was 78 ± 16 (range: 44–100) (Table 1).

**TABLE 1:** Study characteristics

Parameter	*N*	DS (*n* = 24)	Ctrl (*n* = 17)	*p*‐value
Age	41			0.411^b^
Mean (SD)		39 (7)	36 (9)	
Range		27, 53	22, 51	
Sex	41			0.326^a^
Female, *n* (%)		9 (37.5)	9 (52.9)	
Level of ID	19			NA
Mild, *n* (%)		9 (47.4)	NA	
Moderate, *n* (%)		10 (52.6)	NA	

		DS		
Parameter	*N*	Mild ID (*n* = 9)	Moderate ID (*n* = 10)	*p*‐value
Age	19^c^			0.539^b^
Mean (SD)		37.4 (8.9)	39.5 (6.2)	
Range		27, 53	29, 48	
CAMCOG‐DS	19^c^			0.030^b^
Mean (SD)		88 (12)	73 (16)	
Range		60, 100	44, 99	

Abbreviations: CAMCOG‐DS, Cambridge Cognition Examination DS; Ctrl, control; DS, Down syndrome; ID, intellectual disability; SD, standard deviation.

^a^ Pearson's chi‐squared test.

^b^ Wilcoxon rank sum test.

^c^ Three individuals with DS had no CAMCOG‐DS available.

### 3.1 Analysis of retinal vascular changes

#### 3.1.1 Morphological changes

##### Peripapillary vessel number—OCT

When assessing the density plot illustrating the distribution of vessel numbers around the optic disk, the DS group exhibited a higher percentage of eyes with an increased vessel count compared to the control group (Figure S2). This observation was statistically supported by Pearson's chi‐squared test, which revealed that 81.6% of eyes in the DS group had 18 or more detected vessels, in contrast to 39.4% in the control group (*p* < 0.001) (Table 2A). Additionally, GEE analysis predicted an average of 2.5 more vessels in the eyes of individuals with DS compared to control participants (*β* = 2.529, *p* < 0.001) (Table 2B).

**TABLE 2:** Results of the linear regression analysis for retinal vessel count

A	DS (*n* = 38)	Ctrl (*n* = 33)			*p*‐value^*^
	*n* (%)	*n* (%)			**<0.001**
Low (< 18 vessels)	7 (18.4)	20 (60.6)			
High (≥18 vessels)	31 (81.6)	13 (39.4)			

B	Mean (SD)		GEE		
	DS (*n* = 38)	Ctrl (*n* = 33)	*β*	95% CI	*p*‐value
Vessel count	20 (2)	17 (2)	2.529	1.38, 3.67	**<0.001**

*Note*: (A) This table compares the prevalence of eyes in the low and high vessel group between DS and control group. The *p*‐values were calculated using Pearson's Chi‐squared test. (B) This table also presents the results of the linear regression analysis using GEE with vessel count as the outcome and diagnosis as the exposure. *β* coefficient represents the mean change in the retinal vessel count between Ctrl and DS, width Ctrl as the reference group. The model is adjusted for age and sex.

Bolded values are statistically significant.

Abbreviations: CI, confidence interval; Ctrl, control; DS, Down syndrome; GEE, generalized estimating equation; SD, standard deviation.

* Adjusted *p*‐values for multiple comparison using false discovery rate correction.

##### FD—UWF

FD, representing the complexity of the retinal vascular tree, was the highest (more complex) in the posterior pole and lowest in the midperiphery in both groups (Figure S3A and Table 3). When the groups were compared, there was a higher FD in the posterior pole (arterioles: *β* = 0.050, *p* < 0.001; venules: *β* = 0.027, *p* = 0.001) and a lower venular FD in the midperiphery (*β* = −0.029, *p* = 0.047) in DS compared to control (Table 3). The same pattern emerged when the total vascular FD, encompassing arterioles and venules, was evaluated. A higher FD was observed in the posterior pole (*β* = 0.026, *p* < 0.001), while a lower FD was noted in the midperiphery (*β* = −0.021, *p* = 0.030) in association with DS (Table S1).

**TABLE 3:** Results of the linear regression analysis for FD

	Mean (SD)		GEE		
UWF imaging	DS (*n* = 33)	Ctrl (*n* = 34)	*β*	95% CI	*p*‐value^*^
**FD**					
FDa standard	1.14 (0.03)	1.13 (0.02)	0.010	−0.004, 0.023	0.470
FDv standard	1.09 (0.03)	1.10 (0.03)	−0.010	−0.026, 0.007	0.743
FDa posterior	1.39 (0.04)	1.34 (0.04)	0.050	0.026, 0.074	**<0.00**
FDv posterior	1.35 (0.04)	1.32 (0.03)	0.027	0.012, 0.042	**0.001**
FDa midperiphery	1.09 (0.04)	1.10 (0.02)	−0.011	−0.031, 0.010	0.923
FDv midperiphery	1.03 (0.05)	1.06 (0.03)	−0.029	−0.053, −0.006	**0.047**

*Note*: This table presents the results of the linear regression analysis using GEE with FD as the outcome and diagnosis as the exposure. *β* coefficient represents the mean change in the FD between Ctrl and DS, width Ctrl as the reference group. The model is adjusted for age and sex.

Bolded values are statistically significant.

Abbreviations: CI, confidence interval; Ctrl, control; DS, Down syndrome; FD, fractal dimension; FDa, arteriolar fractal dimension; FDR, false discovery rate; FDv, venular fractal dimension; GEE, generalized estimating equation; SD, standard deviation; UWF, ultra widefield.

* Adjusted *p*‐values for multiple comparison using FDR correction.

##### WG, WI, and TORT—UWF

WG, representing the degree of vessel narrowing along the vessel path, showed the highest degree of thinning (larger negative value) in venules across both groups—in the inferotemporal venules in the DS group (−4.96 µm/mm ± 1.91) and the superotemporal venules in the control group (−4.25 µm/mm ± 1.18) (Figure S3B). When comparing the two groups, a lower WG was associated with DS globally (arterioles: *β* = −0.66, *p* = 0.006; venules: *β* = −0.75, *p* = 0.001), as well as in the nasal hemisphere (arterioles: *β* = −0.86, *p* = 0.030) (Table 4). When analyzing individual quadrants, only the inferotemporal arterioles (*β* = −0.92, *p* = 0.001) and venules (*β* = −1.10, *p* = 0.013) showed a significantly higher degree of narrowing in DS compared to the control group (Table S2).

**TABLE 4:** Results of the linear regression analysis for WG, WI, and tortuosity

	Mean (SD)		GEE		
UWF imaging	DS (*n* = 33)	Ctrl (*n* = 34)	*β*	95% CI	*p*‐value^*^
WG (µm/mm)					
WGa global	−3.24 (0.79)	−2.59 (0.90)	−0.66	−1.08, −0.24	**0.006**
WGv global	−4.63 (1.18)	−3.87 (0.70)	−0.75	−1.16, −0.34	**0.001**
WGa temporal	−3.22 (0.89)	−2.74 (0.66)	−0.45	−0.86, −0.04	0.094
WGv temporal	−4.81 (1.74)	−4.06 (0.83)	−0.72	−1.37, −0.07	0.089
WGa nasal	−3.25 (1.27)	−2.44 (1.47)	−0.86	−1.51, −0.21	**0.030**
WGv nasal	−4.44 (1.44)	−3.68 (1.32)	−0.76	−1.45, −0.07	0.092
WI (µm)					
WIa global	113.12 (12.55)	95.58 (10.77)	16.9	10.8, 23.0	**<0.001**
WIv global	143.10 (20.12)	122.75 (12.32)	19.0	10.2, 27.7	**<0.001**
WIa temporal	127.44 (17.36)	107.41 (12.71)	18.3	10.2, 26.4	**<0.001**
WIv temporal	160.71 (25.67)	138.46 (11.89)	20.5	9.94, 31.1	**<0.001**
WIa nasal	98.80 (15.50)	83.74 (14.22)	15.3	7.70, 23.0	**<0.001**
WIv nasal	125.48 (19.24)	107.04 (18.46)	17.6	7.74, 27.5	**<0.001**
Tortuosity					
TORTa global	0.053 (0.051)	0.044 (0.037)	0.006	−0.012, 0.023	0.530
TORTv global	0.055 (0.038)	0.044 (0.042)	0.010	−0.005, 0.025	0.409
TORTa temporal	0.061 (0.063)	0.045 (0.036	0.013	−0.009, 0.036	0.352
TORTv temporal	0.065 (0.051)	0.048 (0.048)	0.014	−0.002, 0.031	0.253
TORTa nasal	0.021 (0.027)	0.029 (0.043)	−0.007	−0.022, 0.007	0.475
TORTv nasal	0.038 (0.050)	0.020 (0.024)	0.016	0.000, 0.033	0.138

*Note*: This table presents the results of the linear regression analysis using GEE with quantitative vascular parameters as the outcome and diagnosis as the exposure. *β* coefficient represents the mean change in the given vascular parameter between Ctrl and DS, width Ctrl as the reference group. The model is adjusted for age and sex.

Bolded values are statistically significant.

Abbreviations: CI, confidence interval; Ctrl, control; DS, Down syndrome; GEE, generalized estimating equation; SD, standard deviation; TORTa, arterial tortuosity; TORTv, venular tortuosity; UWF, ultra widefield; WG, width gradient; WGa, arteriolar width gradient; WGv, venular width gradient; WI, width intercept; WIa, arteriolar width intercept; WIv, venular width intercept.

* Adjusted *p*‐values for multiple comparison using false discovery rate correction.

WI, an estimation of vessel caliber at the optic disc edge and a UWF proxy for parameters originating from conventional fundus image, known as CRAE and CRVE, was found to be higher in venules compared to corresponding arterioles (Figure S3C). Additionally, it was highest in the inferotemporal quadrant in both groups (DS: 169.76 µm ± 27.47; control: 140.64 µm ± 18.96) (Figure S3C). When the two groups were compared, a higher WI associated with DS globally (arterioles: *β* = 16.9, *p* < 0.001; venules: *β* = 19.0, *p* < 0.001), as well as when the temporal (arterioles: *β* = 18.3, *p* < 0.001; venules: *β* = 20.5, *p* < 0.001) and the nasal (arterioles: *β* = 15.3, *p* < 0.001; venules: *β* = 17.6, *p* = 0.001) hemispheres or individual quadrants (*β* = 12.7–27.4, *p* < 0.05) were considered (Table 4, Table S2).

TORT, a measure of the extent of vessel twisting and turning, peaked in the inferotemporal venules in the DS group (0.076 ± 0.067) and the inferotemporal arterioles in the control group (0.056 ± 0.054) (Figure S3D). Comparing the two groups, no significant differences were detected between DS and control (Table 4, Table S2).

#### 3.1.2 Microvascular abnormalities—UWF

A higher number of eyes exhibited microvascular abnormalities (eyes exhibiting microaneurysms, hemorrhages, both) in the DS groups compared to the control group (12 [36.4%] vs. 3 [8.8%]) (Table 5). The microvascular abnormalities were observed almost exclusively in the temporal hemisphere and exclusively in the far‐peripheral zone (data not shown). Comparing the two groups, individuals in the DS group had a significantly higher likelihood of microvascular abnormalities (OR = 8.332, *p* = 0.038) (Table 5). However, when microaneurysms and hemorrhages were analyzed separately using hierarchical phenotyping, no significant differences (*p* > 0.05) were found between DS and controls, potentially due to the small sample size (Table 5).

**TABLE 5:** Results of the logistic regression analysis for microvascular abnormalities

	*n* (%)^a^		GEE		
UWF imaging	DS (*n* = 33)	Ctrl (*n* = 34)	OR	95% CI	*p*‐value^*^
Microvascular abnormality (YES)	12 (36.4)	3 (8.8)	8.332	1.570, 44.21	**0.038**
Microaneurysm (YES)	7 (21.2)	2 (5.9)	5.231	0.993, 27.55	0.153
Haemorrhage (YES)	5 (15.2)	1 (2.9)	8.455	0.880, 81.26	0.191

*Note*: This table presents the results of the GEE‐based logistic regression analysis, with microvascular abnormalities as the outcome and diagnosis as the exposure. The odds ratio (OR) reflects the likelihood of microvascular abnormalities in the DS group compared to the control group (reference). The model is adjusted for age and sex.

Bolded values are statistically significant.

Abbreviations: CI, confidence interval; Ctrl, control; DS, Down syndrome; GEE, generalized estimating equation; UWF, ultra widefield.

^a^ *n* = number of eyes exhibiting microvascular abnormalities and the corresponding percentage.

* Adjusted *p*‐values for multiple comparison using false discovery rate correction.

### 3.2 Analysis of choroidal vascular changes

#### 3.2.1 CVI—OCT

CVI, which represents the proportion of the LA (corresponds to choroidal vessels) to the total choroidal area, was the highest in the central circle of the ETDRS grid in the DS group (41.36% ± 8.55), and the inner ring in the control group (51.71% ± 7.53) (Figure S4 and Table 6). When quadrants of the ETDRS grid were considered, the temporal outer quadrant in DS (44.26% ± 6.98) and the nasal inner quadrant in the control group (51.71% ± 7.53) had the highest proportion of vascular area (Table S3). Comparing the two groups, apart from the nasal outer sector of the ETDRS grid, there was a lower CVI associated with DS in each sector of the ETDRS grid with the greatest difference in the superior outer quadrant (*β* = −12.6, *p* < 0.001) (Table 6 and Table S3).

**TABLE 6:** Results of the linear regression analysis for CVI

	Mean (SD)		GEE		
OCT imaging	DS (*n* = 33)	Ctrl (*n* = 34)	*β*	95% CI	*p*‐value^*^
**CVI (%)**					
Central circle	41.36 (8.55)	50.96 (8.18)	−8.88	−13.5, −4.29	**<0.001**
Inner ring	40.90 (8.30)	51.71 (7.53)	−9.66	−14.0, −5.34	**<0.001**
Outer ring	40.90 (7.51)	50.21 (6.49)	−8.65	−12.4, −4.87	**<0.001**
Global	40.88 (7.32)	50.45 (6.59)	−8.97	−12.7, −5.24	**<0.001**

*Note*: This table presents the results of the linear regression analysis using GEE with CVI as the outcome and diagnosis as the exposure. *β* coefficient represents the mean change in the number of vessels or CVI between Ctrl and DS, with Ctrl as the reference group. The model is adjusted for age and sex.

Bolded values are statistically significant.

Abbreviations: CI, confidence interval; Ctrl, control; CVI, choroidal vascularity index; DS, Down syndrome; GEE, generalized estimating equation; SD, standard deviation.

* Adjusted *p*‐values for multiple comparison using false discovery rate correction.

### 3.3 Relationship between age, sex, and vascular parameters

The effect of age and sex on various retinal and choroidal vascular parameters was assessed by including them as covariates in the multiple regression analysis. Apart from the reduced WIa in the temporal hemisphere that was associated with females compared to males (*β* = −9.99, *p* < 0.018), no age and/or sex showed significant association with the outcome in any of the models (*p* > 0.05).

### 3.4 Relationship between CAMCOG‐DS and vascular parameters in individuals with DS

#### 3.4.1 Relationship between CAMCOG‐DS and quantitative retinal vascular parameters

The only significant relationship between CAMCOG‐DS and quantitative vascular parameters was observed with vascular TORT, showing an inverse association. Specifically, higher CAMCOG‐DS scores were linked to lower global TORTa (*β* = −0.002, *p* = 0.001; marginal *R* ^2^ = 0.310) and lower temporal TORTa (*β* = −0.002, *p* = 0.029 marginal *R* ^2^ = 0.232). For a summary of results, see Supplementary Data.

Models included age and sex as covariates, and many of them revealed significant associations between covariates and retinal vascular parameters in individuals with DS. Lower peripapillary vessel number associated to females compared to males (*β* = −2.47, *p* = 0.010). Lower standard FDa is associated with higher age (*β* = −0.001, *p* = 0.044) and females compared to males (*β* = −0.024, *p* = 0.019). Lower temporal WIa associated with females compared to males (*β* = −21.13, *p* = 0.004). Increased TORTa associated with higher age globally (*β* = 0.002, *p* = 0.004) and temporally (*β* = 0.002, *p* = 0.029). Lower TORT associated with females compared to males globally (TORTa: *β* = −0.050, *p* = 0.006; TORTv: *β* = −0.023, *p* = 0.003), temporally (TORTa: *β* = −0.056, *p* = 0.029; TORTv: *β* = −0.023, *p* = 0.009), and nasally (TORTv: *β* = −0.026, *p* = 0.027). For a summary of results, see Supplementary Data.

#### 3.4.2 Relationship between CAMCOG‐DS and qualitative retinal vascular parameters

Logistic regression analysis using GEE assessing the relationship between microvascular abnormalities and CAMCOG‐DS scores revealed no significant (*p* > 0.05) association. For a summary of results, see Supplementary Data.

#### 3.4.3 Relationship between CAMCOG‐DS and choroidal vascular parameters

Logistic regression analysis using GEE assessing the relationship between CVI and CAMCOG‐DS scores revealed no significant (*p* > 0.05) association. For a summary of results, see Supplementary Data.

## 4 DISCUSSION

Identifying early, noninvasive, and inexpensive biomarkers for proxy outcome measures is crucial for the success of AD trials, especially for those with DS, as many are likely to benefit from early treatment trials to prevent the onset of AD pathology. Individuals with DS are genetically predisposed to develop AD at an earlier age compared to people with no DS. By the age of 40, all individuals with DS exhibit cerebral AD pathology due to their genetic predisposition caused by the trisomy of chromosome 21.^19^ Given the mean age and age range of our cohort, this makes it a valuable group for investigating potential early ocular biomarkers of AD that may reflect early cerebrovascular changes. Retinal and choroidal vascular changes have been proposed as early biomarkers for AD in the typically developing population (sAD). Imaging the retina is quick, inexpensive, well tolerated, and capable of achieving much higher resolution than currently available brain imaging.

Our findings regarding the number of vessels around the optic disk are consistent with previous studies,^9^, ^10^, ^23^, ^30^, ^31^, ^32^ which have also identified supernumerary vessels in individuals with DS. These additional vessels give the posterior pole a distinctive spoke‐like appearance. Spoke‐like vessel patterns are characteristic of the earliest stages of human embryonic retinal development and typically mature into more refined structures over time.^33^ The persistence of these structures in DS may suggest altered retinal development during embryogenesis and might be linked to endostatin and Dyrk1A, proteins encoded on chromosome 21, that can affect angiogenesis and vessel branching.^11^, ^34^

We also observed wider vessel calibers at the edge of the optic disk. There is no other published research on this specific aspect of retinal vasculature in the DS population, although a previous study reported wider vessels in mouse models of DS.^35^ Narrower retinal arterioles and wider venules on conventional fundus images have been linked to increased dementia risk in typically developing individuals, reflecting potential cerebrovascular changes.^36^ While narrower calibers have been associated with sAD using conventional fundus imaging,^4^, ^5^, ^37^ Joseph et al. found lower arteriolar and venular calibers linked to MCI in typically developing individuals using the same method as our study.^3^ In contrast, we observed wider arterioles in DS, possibly also influenced by factors like lower systemic blood pressure in this population.^38^ Our findings may reflect cerebrovascular alterations in DS potentially due to the developing AD pathology, but this connection requires further exploration through studies combining retinal and brain imaging.

In addition to the wider vessels at the edge of the optic disk, we found that these vessels thin along their path more rapidly in individuals with DS than in controls, as indicated by the lower WG. This aspect of retinal vasculature has not been previously explored in this patient population. However, faster narrowing has been reported in individuals with MCI and sAD.^1^, ^2^ The WG is a relatively new vascular parameter that has yet to be widely studied. Its relationship to brain changes, particularly in cerebral vasculature, remains unexplored.

FD, a measure of retinal vascular complexity, has been underexplored in DS. Our study observed increased FD at the posterior pole (near the optic disk) and decreased FD in the mid‐periphery. The posterior increase suggests a denser vascular network, potentially due to supernumerary vessels and wider calibers, while the peripheral decrease may reflect faster vessel thinning in DS. In contrast, FD typically decreases at the posterior pole in sAD using conventional fundus imaging.^4^, ^37^, ^39^ Few studies have examined peripheral retinal FD in sAD using UWF imaging and our methodology, yielding conflicting results.^1^, ^2^, ^3^ As the first study assessing FD in DS, our findings highlight key differences and warrant further investigation.

Vascular TORT is influenced by factors such as genetics, degenerative vascular disease, and changes in blood flow and pressure, which can alter vessel wall properties.^40^ While TORT is a debated parameter in dementia research, findings are inconsistent: some studies report lower arteriolar TORT in sAD,^37^ others find no differences ^41^ or increased TORT in both arterioles and venules.^5^ These discrepancies may arise from variations in imaging techniques (conventional vs. UWF imaging), ROIs, and TORT measurement methods. This study is the first to assess vascular TORT in DS using UWF imaging, revealing no differences compared to control. However, arterial TORT was the only vascular parameter in our study that showed an inverse relationship with CAMCOG‐DS scores. This is an interesting finding and underscoring the need to investigate this parameter in this population further.

We observed a higher prevalence of microvascular abnormalities (a combination of microaneurysms and hemorrhages) exclusively in the far‐peripheral retina of individuals with DS. Peripheral microvascular abnormalities are commonly associated with diabetes.^42^ However, we ensured that participants with diabetes were excluded from our study. We are unaware of studies exploring similar changes in the retinal periphery in relation to the AD continuum in DS or the typically developing population. Most importantly, recent research has shown a higher number of cerebral microbleeds, predominantly located in the cerebellum, occipital lobe, and temporal lobes, are associated with adults with DS, independent of vascular risk factors, such as hypertension, diabetes, and dyslipidemia.^43^ This finding had been associated with cerebral amyloid angiopathy (CAA), often observed in the same brain regions in AD.^43^ Therefore, we hypothesize that the microvascular abnormalities detected in the far‐peripheral retina in our DS cohort may result from blood vessel damage caused by amyloid angiopathy. *Post mortem* studies have shown the presence of amyloid in the blood vessels of retinas from sAD donors,^16^, ^44^ but we are unaware of *post mortem* studies in the retinas of individuals with DS. An exploratory study by Rafii et al. used a modified SLO and identified amyloid‐like deposits in vivo in DS retinas^45^ suggesting that further research is needed to establish the CAA hypothesis in the retina of individuals with DS as these could become biomarkers for patient stratification in clinical trials. This is crucial as individuals with CAA are more susceptible to treatment‐related side effects due to the increased fragility of their blood vessels (amyloid‐related imaging abnormalities‐hemorrhage [ARIA‐H]).^46^

In the typically developing population, reduced CVI, which measures the ratio of vascular area to total choroidal area, has been associated with MCI.^7^ Our study found a lower CVI in individuals with DS compared to controls. This is the first investigation assessing CVI in DS. Our previous research revealed increased choroidal thickness in adults with DS, suggesting that the combination of thicker choroids and reduced CVI may indicate underlying pathological changes, including inflammation, potentially linked to the onset of dementia.^12^, ^19^ While no human studies observe amyloid *β* in the choroid, amyloid accumulation in the choroidal vasculature has been reported in transgenic mice and rat models of AD.^17^, ^18^ We propose that the lower CVI could result from inflammation and vascular damage caused by amyloid angiopathy. Future studies are warranted to investigate the presence of amyloid in the choroidal vasculature in dementia in both DS and typically developing populations.

When assessing the relationship between CAMCOG‐DS and vascular parameters, only arterial TORT showed a significant inverse association, as previously discussed. In these models, age and sex were included as covariates, revealing significant associations with many vascular parameters. This underscores the importance of controlling for these factors when evaluating the utility of retinal vascular biomarkers in DS.

It is important to note that many of the studies mentioned above differ in sample sizes and methodologies for measuring retinal vascular parameters, often yielding conflicting findings and underscoring the need for standardized protocols.

Our study had several limitations. Although our DS population is well positioned, in terms of age range, to assess signs of early AD pathology,^19^ we do not have direct evidence of cerebral AD pathology within our cohort. We had a small cohort size without information on axial length and refractive errors, which potentially could affect retinal vascular measurements.^47^ We cannot rule out that the decreased CVI observed in our DS cohort may be related to the lower thyroid hormone levels commonly found in DS, which can increase extracellular matrix components in the choroid.^48^, ^49^ Comorbidities like Moyamoya disease–a cerebrovascular disorder affecting the internal carotid arteries more prevalent in DS—although ocular manifestation is uncommon, can lead to retinal microvascular changes such as hemorrhaging.^50^ Furthermore, systemic vascular risk factors, such as hypertension, diabetes, and dyslipidemia, could affect retinal and choroidal vasculature. While we could exclude participants with diabetes, data on other vascular risk factors were lacking. Further research is crucial to understanding how retinal vascular parameters change over time in individuals with DS, taking into account the important confounders listed above.

In summary, our DS cohort exhibited greater thinning of retinal vessels along their path, reduced complexity of the vascular tree in the peripheral retina, and lower choroidal vascularity—changes that mirror those observed in individuals with MCI and AD in typically developing population.^2^, ^7^ Notably, the increased number of microvascular abnormalities (microaneurysms and hemorrhages) in the peripheral retina, potentially linked to amyloid angiopathy, has not yet been reported in AD. Given that our DS cohort is at an age where most individuals have developed cerebral AD pathology, we propose that eye imaging provides valuable insight as a promising, non‐invasive, and cost‐effective tool for identifying early AD biomarkers in DS, potentially reflecting cerebrovascular changes. Further research with standardized methodologies, larger sample sizes, a broader age range, and *post mortem* validation is essential to confirm these retinal markers and their utility in clinical trials for DS.

## CONFLICT OF INTEREST STATEMENT

Jamie Mitchell‐Unrestricted PhD studentship supplement from Optos Plc. Adam Threlfall‐Funded by Optos Plc. Kenneth Sloan–No funding to disclose. Luke Smyth–No funding to disclose. Jessica Beresford‐Webb–No funding to disclose. Madeleine J. Walpert–No funding to disclose. Tunde Peto‐Unrestricted educational grant from Optos Plc. Tom MacGillivray–No funding to disclose. Anthony Holland‐Trustee of the UK Down Syndrome Association (unpaid). Imre Lengyel‐Unrestricted research funding from Optos Plc. Lajos Csincsik‐Unrestricted PhD studentship from Optos Plc. Author disclosures are available in Supporting Information.

## CONSENT STATEMENT

The study received ethical approval from the East of England Cambridge Central Research Ethics Committee (study ref.^14^/EE/1118) and was conducted in accordance with the World Medical Association Declaration of Helsinki. Written informed consent was obtained from all participants. For individuals with DS who lacked the capacity to consent, advice was sought from an identified consultee in accordance with the Mental Capacity Act 2005.

## Supporting information



Supporting Information

Supporting Information

Supporting Information
