# Supplementary material for: Choroidal and retinal vascular changes in adults with Down syndrome: Insights into the Alzheimer's disease continuum
Source: Alzheimers Dement. 2025 May 12;21(5):e70228. doi: 10.1002/alz.70228 (PMC12069005; doi:10.1002/alz.70228)
Supplement: Supplementary file 2 — Supporting Information [file ALZ-21-e70228-s002.pdf]

## Supplementary file 1

**Supplementary tables:**

**Table S1:** Results of the linear regression analysis for fractal dimension

| **UWF imaging** | **DS** (n=33) | **Ctrl** (n=34) | **GEE** | | |
|---|---|---|---|---|---|
| | **Mean (SD)** | | **Beta** | **95% CI** | **P-value*** |
| **Fractal dimension** | | | | | |
| FD standard | 1.18 (0.02) | 1.18 (0.01) | -0.002 | -0.011, 0.006 | 0.649 |
| FD posterior | 1.49 (0.02) | 1.47 (0.02) | 0.026 | 0.014, 0.039 | **0.000** |
| FD midperiphery | 1.13 (0.03) | 1.15 (0.01) | -0.021 | -0.037, -0.005 | **0.030** |

This table presents the results of the linear regression analysis using GEE with total vascular fractal dimension (encompassing both arterioles and venules) as the outcome and diagnosis as the exposure. Beta coefficient represents the mean change in the fractal dimension between Ctrl and DS, width Ctrl as the reference group. The model is adjusted for age and sex. Bolded values are statistically significant. Abbreviations: FD, fractal dimension; SD, Standard deviation; DS, Down Syndrome; Ctrl, Control; CI, Confidence interval; *adjusted P-values for multiple comparison using false discovery rate correction;

**Tables S2:** Results of the linear regression analysis for width gradient, width intercept and tortuosity

| UWF imaging | **DS** (n=33) | **Ctrl** (n=34) | **GEE** | | |
|---|---|---|---|---|---|
| | **Mean (SD)** | | **Beta** | **95% CI** | **P-value*** |
| **Width gradient (μm/mm)** | | | | | |
| WGa ST | -2.81 (1.66) | -2.74 (0.76) | 0.01 | -0.65, 0.66 | 0.984 |
| WGv ST | -4.65 (2.42) | -4.25 (1.18) | -0.34 | -1.21, 0.53 | 0.478 |
| WGa IT | -3.64 (1.05) | -2.74 (1.00) | -0.92 | -1.42, -0.41 | **0.001** |
| WGv IT | -4.96 (1.91) | -3.87 (1.01) | -1.10 | -1.85, -0.35 | **0.013** |
| WGa IN | -3.51 (2.21) | -2.51 (1.55) | -1.01 | -2.00, -0.02 | 0.136 |
| WGv IN | -4.14 (1.85) | -3.43 (1.51) | -0.62 | -1.43, 0.19 | 0.291 |
| WGa SN | -2.99 (1.96) | -2.36 (2.40) | -0.74 | -1.73, 0.25 | 0.297 |

| | | | | | |
|---|---|---|---|---|---|
| WGv SN | -4.75 (2.27) | -3.92 (2.03) | -0.92 | -2.06, 0.22 | 0.343 |
| **Width intercept (µm)** | | | | | |
| WIa ST | 122.54 (26.84) | 107.21 (12.74) | 12.7 | 2.98, 22.3 | **0.016** |
| WIv ST | 151.67 (31.71) | 136.28 (13.01) | 13.9 | 2.94, 24.8 | **0.039** |
| WIa IT | 132.34 (16.97) | 107.61 (17.02) | 24.4 | 15.3, 33.5 | **0.000** |
| WIv IT | 169.76 (27.47) | 140.64 (18.96) | 27.4 | 14.3, 40.5 | **0.000** |
| WIa IN | 98.53 (21.93) | 83.33 (14.13) | 15.6 | 6.15, 25.0 | **0.004** |
| WIv IN | 123.25 (24.92) | 103.74 (18.00) | 17.4 | 5.68, 29.1 | **0.011** |
| WIa SN | 99.06 (24.23) | 84.16 (21.28) | 15.1 | 4.47, 25.7 | **0.016** |
| WIv SN | 127.71 (24.84) | 110.34 (27.57) | 18.0 | 5.22, 30.8 | **0.017** |
| **Tortuosity** | | | | | |
| TORTa ST | 0.040 (0.053) | 0.032 (0.031) | 0.006 | -0.012, 0.023 | 0.812 |
| TORTv ST | 0.050 (0.062) | 0.047 (0.068) | -0.001 | -0.023, 0.021 | 0.928 |
| TORTa IT | 0.060 (0.058) | 0.056 (0.054) | 0.005 | -0.020, 0.029 | 0.706 |
| TORTv IT | 0.076 (0.067) | 0.047 (0.052) | 0.026 | 0.001, 0.051 | 0.121 |
| TORTa IN | 0.019 (0.031) | 0.017 (0.020) | 0.003 | -0.010, 0.015 | 0.922 |
| TORTv IN | 0.032 (0.041) | 0.026 (0.050) | 0.006 | -0.013, 0.026 | 0.910 |
| TORTa SN | 0.019 (0.038) | 0.026 (0.036) | -0.006 | -0.022, 0.009 | 0.813 |
| TORTv SN | 0.030 (0.065) | 0.019 (0.029) | 0.007 | -0.013, 0.027 | 0.697 |

This table presents the results of the linear regression analysis using GEE with vascular parameters as the outcome and diagnosis as the exposure. Beta coefficient represents the mean change in the given vascular parameter between Ctrl and DS, width Ctrl as the reference group. The model is adjusted for age and sex. Bolded values are statistically significant. Abbreviations: Abbreviations: DS, Down Syndrome; Ctrl, Control; WGa, arteriolar width gradient; WGv, venular width gradient; WIa, arteriolar width intercept; WIv, venular width intercept; TORTa, arterial tortuosity; TORTv, venular tortuosity; ST, superotemporal; IT, inferotemporal; IN, inferonasal; SN, superonasal; SD, standard deviation; CI, confidence interval; *adjusted P-values for multiple comparison using false discovery rate correction;

**Table S3**: Results of the linear regression analysis for choroidal vascularity index

| OCT imaging | DS (n=30) | Ctrl (n=34) | GEE | | |
|---|---|---|---|---|---|
| | **Mean (SD)** | | **Beta** | **95% CI** | **P value*** |
| **CVI (%)** | | | | | |
| Central circle | 41.36 (8.55) | 50.96 (8.18) | -8.88 | -13.5, -4.29 | **0.000** |
| Superior inner | 38.27 (10.45) | 50.62 (7.84) | -11.1 | -16.4, -5.87 | **0.000** |
| Temporal inner | 41.92 (7.10) | 51.41 (7.66) | -9.20 | -13.3, -5.08 | **0.000** |
| Inferior inner | 41.04 (8.01) | 50.79 (8.79) | -9.81 | -14.7, -4.92 | **0.000** |
| Nasal inner | 40.90 (8.30) | 51.71 (7.53) | -9.66 | -14.0, -5.34 | **0.000** |
| Inner ring | 40.77 (7.52) | 51.14 (7.56) | -9.99 | -14.1, -5.92 | **0.000** |
| Superior outer | 36.74 (10.97) | 50.37 (6.41) | -12.6 | -17.5, -7.68 | **0.000** |
| Temporal outer | 44.26 (6.98) | 51.58 (6.60) | -7.28 | -11.1, -3.42 | **0.001** |
| Inferior outer | 39.21 (9.91) | 50.07 (9.07) | -9.75 | -14.8, -4.66 | **0.000** |
| Nasal outer | 43.35 (8.68) | 48.86 (6.99) | -4.92 | -9.24, -0.61 | 0.076 |
| Outer ring | 40.90 (7.51) | 50.21 (6.49) | -8.65 | -12.4, -4.87 | **0.000** |
| Global | 40.88 (7.32) | 50.45 (6.59) | -8.97 | -12.7, -5.24 | **0.000** |

This table presents the results of the linear regression analysis using GEE with CVI as the outcome and diagnosis as the exposure. Beta coefficient represents the mean change in CVI between Ctrl and DS, with Ctrl as the reference group. The model is adjusted for age and sex. Bolded values are statistically significant. Abbreviations: DS, Down Syndrome; Ctrl, Control, SD, standard deviation, CI, confidence interval; *adjusted P-values for multiple comparison using false discovery rate correction;

**Supplementary Figures:**

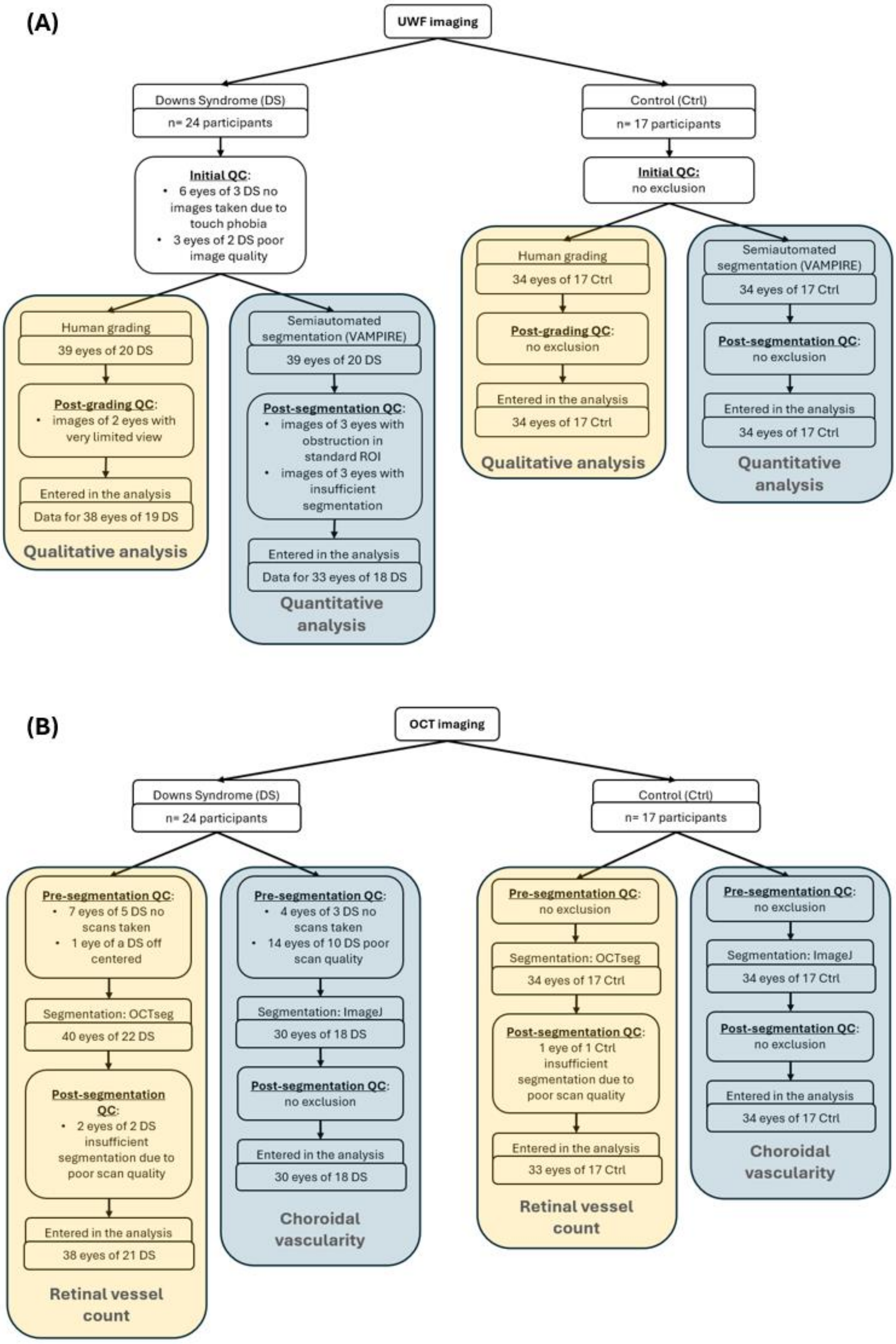


**Figure S1** Imaging modality quality control

Flow chart illustrating the inclusion and exclusion criteria at various quality control (QC) steps for the UWF and OCT pipelines. Abbreviations: UWF, ultra-widefield; OCT, optical coherence tomography; QC, quality control; ROI, region of interest; DS, Down syndrome; Ctrl, control;

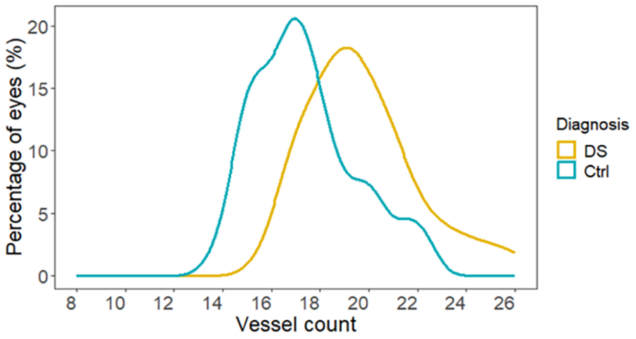


**Figure S2** Vessel Count

The density plot illustrates the distribution of vessel numbers segmented on optic nerve head scans (with a 3.4 mm diameter ring centred on the disk) using OCTseg software, in eyes from the DS group (yellow) and the control group (blue). Abbreviations: DS, Down syndrome; Ctrl, control.

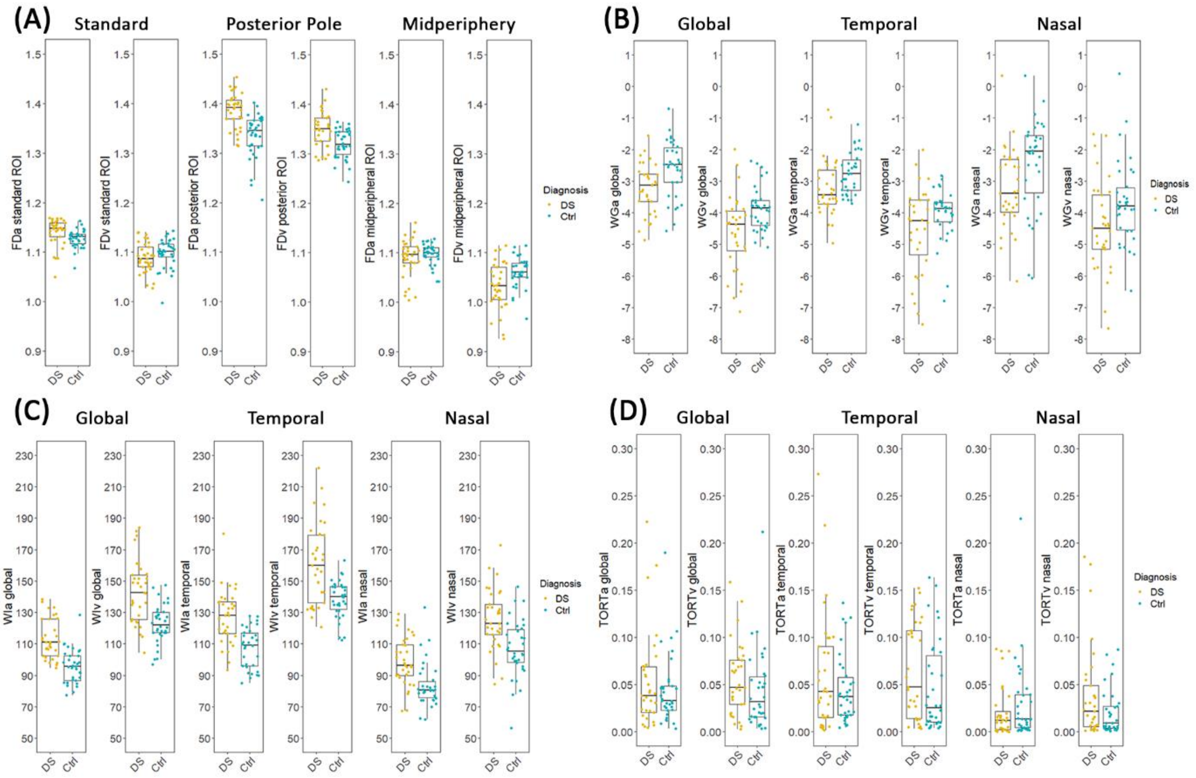


**Figure S3** Quantitative retinal vasculature parameters

Boxplots showing the quantitative retinal vascular parameters extracted from ultra-widefield images in DS (yellow) and control (blue). Abbreviations: DS, Down syndrome; Ctrl, control; FDa, arteriolar fractal dimension; FDv, venular fractal dimension; WGa, arteriolar width gradient; WGv, venular width gradient; WIa, arteriolar width intercept; WIv, venular width intercept; TORTa, arteriolar tortuosity; TORTv, venular tortuosity;

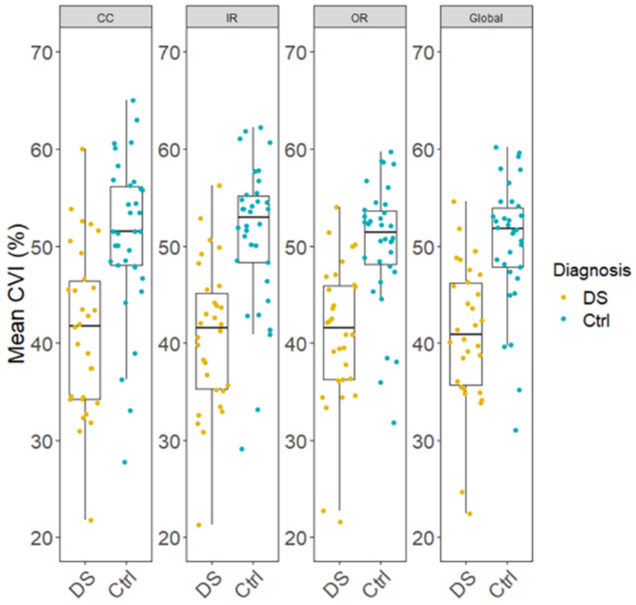


**Figure S4** Mean Choroidal Vascularity Index

Boxplots (E) showing the mean CVI values for the central circle, inner ring, and outer ring of the ETDRS grid, as well as the global mean CVI values (entire ETDRS grid) in DS (yellow) and control (blue) participants. Boxplots with the mean CVI values for each quadrant of the inner and outer rings are provided in the supplementary document. Abbreviations: DS, Down syndrome; Ctrl, control; CVI, choroidal vascularity index; CC, central circle; IR, inner ring; OR, outer ring;
