# Supplementary material for: Choroidal and retinal vascular changes in adults with Down syndrome: Insights into the Alzheimer's disease continuum
Source: Alzheimers Dement. 2025 May 12;21(5):e70228. doi: 10.1002/alz.70228 (PMC12069005; doi:10.1002/alz.70228)
Supplement: Supplementary file 3 — Supporting Information [file ALZ-21-e70228-s003.pdf]

Supplementary file 2

Peripapillary vessel number vs CAMCOG-DS

Variable	N	Beta	95% CI[1]	p-value	q-value[2]
**CAMCOG**	32	-0.019	-0.066, 0.028	0.429	0.643
**Age**	32	-0.010	-0.109, 0.090	0.852	0.852
**Sex**					
M	23	—	—		
F	9	-2.469	-4.120, -0.818	**0.003**	**0.010**

[1] CI = Confidence Interval

[2] False discovery rate correction for multiple testing

Quantitative vascular parameters vs CAMCOG-DS

Fractal (FD)
Width Gradient (WG)
Width Intercept (WI)
Tortuosity (TORT)

	FDa standard					FDa posterior					FDa midperipheral				
**Variable**	**N**	**Beta**	**95% CI[1]**	**p-value**	**q-value[2]**	**N**	**Beta**	**95% CI[1]**	**p-value**	**q-value[2]**	**N**	**Beta**	**95% CI[1]**	**p-value**	**q-value[2]**
**CAMCOG**	31	-0.001	-0.002, 0.000	0.126	0.126	31	-0.001	-0.002, 0.000	0.063	0.099	31	-0.001	-0.002, 0.001	0.408	0.595
**Age**	31	-0.001	-0.002, 0.000	**0.029**	**0.044**	31	-0.001	-0.003, 0.001	0.319	0.319	31	0.000	-0.002, 0.001	0.595	0.595
**Sex**															
M	22	—	—			22	—	—			22	—	—		
F	9	-0.024	-0.041, -0.007	**0.006**	**0.019**	9	-0.024	-0.049, 0.002	0.066	0.099	9	-0.026	-0.058, 0.005	0.104	0.311

[1]CI = Confidence Interval

[2]False discovery rate correction for multiple testing

	FDv standard					FDv posterior					FDv midperipheral				
**Variable**	**N**	**Beta**	**95% CI[1]**	**p-value**	**q-value[2]**	**N**	**Beta**	**95% CI[1]**	**p-value**	**q-value[2]**	**N**	**Beta**	**95% CI[1]**	**p-value**	**q-value[2]**
**CAMCOG**	31	0.000	-0.001, 0.001	0.809	0.809	31	0.000	-0.001, 0.000	0.480	0.480	31	0.000	-0.001, 0.001	0.336	0.934
**Age**	31	-0.001	-0.002, 0.001	0.506	0.809	31	-0.001	-0.002, 0.001	0.260	0.390	31	0.000	-0.003, 0.003	0.885	0.934
**Sex**															
M	22	—	—			22	—	—			22	—	—		
F	9	-0.004	-0.030, 0.022	0.751	0.809	9	-0.027	-0.050, -0.003	**0.026**	0.078	9	-0.001	-0.037, 0.034	0.934	0.934

[1]CI = Confidence Interval

[2]False discovery rate correction for multiple testing

	WGa global					WGa temporal					WGa nasal				
**Variable**	**N**	**Beta**	**95% CI**[1]	**p-value**	**q-value**[2]	**N**	**Beta**	**95% CI**[1]	**p-value**	**q-value**[2]	**N**	**Beta**	**95% CI**[1]	**p-value**	**q-value**[2]
**CAMCOG**	31	-0.006	-0.019, 0.006	0.323	0.484	31	0.008	-0.015, 0.032	0.483	0.709	31	-0.020	-0.041, 0.000	0.053	0.159
**Age**	31	0.002	-0.040, 0.044	0.933	0.933	31	-0.008	-0.049, 0.033	0.709	0.709	31	0.012	-0.065, 0.089	0.759	0.759
**Sex**															
M	22	—	—			22	—	—			22	—	—		
F	9	0.426	-0.055, 0.906	0.083	0.248	9	0.492	-0.169, 1.153	0.145	0.434	9	0.353	-0.427, 1.133	0.375	0.562

[1]CI = Confidence Interval

[2]False discovery rate correction for multiple testing

	WGv global					WGv temporal					WGv nasal				
**Variable**	**N**	**Beta**	**95% CI**[1]	**p-value**	**q-value**[2]	**N**	**Beta**	**95% CI**[1]	**p-value**	**q-value**[2]	**N**	**Beta**	**95% CI**[1]	**p-value**	**q-value**[2]
**CAMCOG**	31	-0.008	-0.053, 0.038	0.748	0.748	31	-0.010	-0.086, 0.066	0.792	0.792	31	-0.005	-0.035, 0.025	0.756	0.958
**Age**	31	0.038	-0.030, 0.105	0.277	0.748	31	0.038	-0.064, 0.140	0.464	0.792	31	0.037	-0.024, 0.099	0.235	0.705
**Sex**															
M	22	—	—			22	—	—			22	—	—		
F	9	0.180	-0.904, 1.265	0.744	0.748	9	0.388	-1.418, 2.195	0.673	0.792	9	-0.027	-1.039, 0.985	0.958	0.958

[1]CI = Confidence Interval

[2]False discovery rate correction for multiple testing

	Wla global					Wla temporal					Wla nasal				
**Variable**	**N**	**Beta**	**95% CI[1]**	**p-value**	**q-value[2]**	**N**	**Beta**	**95% CI[1]**	**p-value**	**q-value[2]**	**N**	**Beta**	**95% CI[1]**	**p-value**	**q-value[2]**
**CAMCOG**	31	-0.100	-0.589, 0.390	0.690	0.690	31	-0.546	-1.106, 0.014	0.056	0.084	31	0.355	-0.138, 0.848	0.158	0.373
**Age**	31	-0.231	-0.877, 0.415	0.483	0.690	31	0.054	-0.700, 0.807	0.889	0.889	31	-0.486	-1.311, 0.340	0.249	0.373
**Sex**															
M	22	—	—			22	—	—			22	—	—		
F	9	-12.81	-24.09, -1.523	**0.026**	0.078	9	-21.13	-33.91, -8.345	**0.001**	**0.004**	9	-4.141	-16.68, 8.399	0.518	0.518

[1]CI = Confidence Interval

[2]False discovery rate correction for multiple testing

	Wlv global					Wlv temporal					Wlv nasal				
**Variable**	**N**	**Beta**	**95% CI[1]**	**p-value**	**q-value[2]**	**N**	**Beta**	**95% CI[1]**	**p-value**	**q-value[2]**	**N**	**Beta**	**95% CI[1]**	**p-value**	**q-value[2]**
**CAMCOG**	31	0.157	-0.648, 0.962	0.702	0.702	31	0.343	-0.759, 1.445	0.542	0.542	31	-0.026	-0.604, 0.552	0.930	0.930
**Age**	31	-0.637	-1.651, 0.377	0.218	0.330	31	-0.803	-2.183, 0.577	0.254	0.497	31	-0.445	-1.263, 0.373	0.287	0.430
**Sex**															
M	22	—	—			22	—	—			22	—	—		
F	9	-12.50	-32.47, 7.476	0.220	0.330	9	-13.47	-40.64, 13.70	0.331	0.497	9	-11.28	-27.36, 4.800	0.169	0.430

[1]CI = Confidence Interval

[2]False discovery rate correction for multiple testing

	torta global					torta temporal					torta nasal				
**Variable**	**N**	**Beta**	**95% CI[1]**	**p-value**	**q-value[2]**	**N**	**Beta**	**95% CI[1]**	**p-value**	**q-value[2]**	**N**	**Beta**	**95% CI[1]**	**p-value**	**q-value[2]**
**CAMCOG**	31	-0.002	-0.003, -0.001	**0.000**	**0.001**	31	-0.002	-0.004, 0.000	**0.021**	**0.029**	31	-0.002	-0.005, 0.001	0.267	0.464
**Age**	31	0.002	0.001, 0.004	**0.003**	**0.004**	31	0.002	0.000, 0.004	**0.027**	**0.029**	31	0.001	-0.002, 0.004	0.464	0.464
**Sex**															
M	22	—	—			22	—	—			22	—	—		
F	9	-0.050	-0.085, -0.014	**0.006**	**0.006**	9	-0.056	-0.107, -0.006	**0.029**	**0.029**	9	-0.040	-0.119, 0.038	0.311	0.464

[1]CI = Confidence Interval

[2]False discovery rate correction for multiple testing

	tortv global					tortv temporal					tortv nasal				
**Variable**	**N**	**Beta**	**95% CI[1]**	**p-value**	**q-value[2]**	**N**	**Beta**	**95% CI[1]**	**p-value**	**q-value[2]**	**N**	**Beta**	**95% CI[1]**	**p-value**	**q-value[2]**
**CAMCOG**	31	0.000	-0.001, 0.001	0.574	0.574	31	0.000	-0.001, 0.000	0.350	0.350	31	0.000	-0.001, 0.001	0.847	0.847
**Age**	31	0.001	0.000, 0.001	0.066	0.099	31	0.001	0.000, 0.002	0.256	0.350	31	0.001	0.000, 0.002	0.111	0.167
**Sex**															
M	22	—	—			22	—	—			22	—	—		
F	9	-0.023	-0.037, -0.009	**0.001**	**0.003**	9	-0.023	-0.040, -0.006	**0.009**	**0.026**	9	-0.026	-0.045, -0.006	**0.009**	**0.027**

[1]CI = Confidence Interval

[2]False discovery rate correction for multiple testing

Qualitative vascular parameters vs CAMCOG-DS

Microvascular abnormality

Microaneurysm

Haemorrhage

	Microvascular abnormality					Microaneurysm					Haemorrhage				
**Variable**	**N**	**OR**[1]	**95% CI**[1]	**p-value**	**q-value**[2]	**N**	**OR**[1]	**95% CI**[1]	**p-value**	**q-value**[2]	**N**	**OR**[1]	**95% CI**[1]	**p-value**	**q-value**[2]
**CAMCOG**	31	0.968	0.911, 1.030	0.305	0.401	31	0.964	0.912, 1.019	0.200	0.300	31	0.962	0.874, 1.059	0.434	0.853
**Age**	31	0.958	0.867, 1.059	0.401	0.401	31	0.932	0.860, 1.009	0.082	0.246	31	1.003	0.843, 1.193	0.977	0.977
**Sex**															
M	22	—	—			22	—	—			22	—	—		
F	9	2.173	0.532, 8.886	0.280	0.401	9	1.540	0.331, 7.166	0.582	0.582	9	1.773	0.247, 12.72	0.569	0.853

[1]OR = Odds Ratio, CI = Confidence Interval

[2]False discovery rate correction for multiple testing

CVI vs CAMCOG-DS

	CVI CC					CVI IR					CVI OR					CVI Global				
**Variable**	**N**	**Beta**	**95% CI[1]**	**p-value**	**q-value[2]**	**N**	**Beta**	**95% CI[1]**	**p-value**	**q-value[2]**	**N**	**Beta**	**95% CI[1]**	**p-value**	**q-value[2]**	**N**	**Beta**	**95% CI[1]**	**p-value**	**q-value[2]**
**CAMCOG**	25	0.04	-0.11, 0.19	0.610	0.610	25	0.06	-0.07, 0.20	0.341	0.512	25	0.08	-0.06, 0.23	0.261	0.261	25	0.08	-0.06, 0.22	0.280	0.358
**Age**	25	-0.17	-0.44, 0.10	0.226	0.610	25	-0.15	-0.32, 0.02	0.083	0.250	25	-0.29	-0.61, 0.04	0.085	0.254	25	-0.25	-0.53, 0.03	0.079	0.237
**Sex**																				
M	16	—	—			16	—	—			16	—	—			16	—	—		
F	9	1.70	-4.31, 7.71	0.579	0.610	9	-1.22	-5.56, 3.12	0.582	0.582	9	-2.20	-6.02, 1.62	0.259	0.261	9	-1.81	-5.66, 2.05	0.358	0.358

[1]CI = Confidence Interval

[2]False discovery rate correction for multiple testing
